# Health worker transfer processes within the public health sector in Ghana: a study of three districts in the Eastern Region

**DOI:** 10.1186/s12960-019-0379-z

**Published:** 2019-06-24

**Authors:** A. C. S. Heerdegen, M. Bonenberger, M. Aikins, P. Schandorf, P. Akweongo, K. Wyss

**Affiliations:** 1 Swiss Centre for International Health, Swiss Tropical and Public Health Institute, Socinstrasse 57, P.O. Box, CH-4002, Basel, Switzerland; 2 University of Basel, Petersplatz 1, P.O. Box, CH-4001, Basel, Switzerland; 3 FAIRMED, Aarbergergasse 29, P.O. Box, 3001, Bern, Switzerland; 4 School of Public Health, College of Health Sciences, University of Ghana, P. O. Box LG13, Accra, Legon Ghana; 5 Nursing and Midwifery Training College, P. O. Box KF 142, Koforidua, Eastern Region Ghana

## Abstract

**Introduction:**

The lack of appropriate policies and procedures to ensure transparent transfer practices is an important source of dissatisfaction among health workers in low- and middle-income countries. In order to alter and improve current practices, a more in-depth and context-specific understanding is needed. This study aims to (1) identify rationales behind transfer decisions in Ghana and (2) examine how transfers are managed in practice versus in policies.

**Methods:**

The study took place in 2014 in three districts in Eastern Ghana. The study population included (1) national, regional, and district health administrators with decision-making authority in terms of transfer decisions and (2) health workers who had transferred between 2011 and 2014. Data was collected through semi-structured and structured face-to-face interviews focusing on rationales behind transfer decisions, health administrators’ role in managing transfers, and health workers’ experience of transfers. A data triangulation approach was applied to compare identified practices with national policies and procedures.

**Results:**

A total of 44 health workers and 21 administrators participated in the study. Transfers initiated by health workers were mostly based on family conditions and preferences to move away from rural areas, while transfers initiated by administrators were based on service requirements, productivity, and performance. The management of transfers was not guided by clear and explicit procedures and thus often depended on the discretion of decision-makers. Moreover, health workers frequently reported not being involved in transfer decision-making processes. We found existing staff perceptions of a non-transparent system.

**Conclusion:**

Our findings suggest a need to foster incentives to attract and retain health workers in rural areas. Moreover, health worker-centered procedures and systems that effectively guide and monitor transfer practices must be developed to ensure that transfers are carried out in a timely, fair, and transparent way.

## Introduction

In low- and middle-income countries (LMICs), posting and transfer (PT) practices, referring to how frontline health workers and administrators are geographically posted and transferred within public health facilities, have been recognized as a main barrier for having an effective workforce [1–3]. Previous studies found that health workers’ dissatisfaction with their postings and inadequate support for them to discharge their roles effectively are linked to absenteeism, low morale, and poor quality of health services [4–6].

In Ghana, as well as in other LMICs, policies and procedures to guide PT decisions exist, yet they have been described as being ambiguous, arbitrary, and non-transparent in practice [4, 6–8]. Due to a lack of clear guidelines and procedures, PT decisions are likely to be formally or informally negotiated outcomes based on diverging interests from the ones posting or transferring and the ones being posted or transferred [2, 6, 7].

The healthcare system in Ghana is administratively organized at the national, regional, and district level [9]. The national level, Ghana Health Service (GHS) headquarters, posts newly graduated health professionals to the ten regions in Ghana, each of which are headed by a regional health administration (RHA). The RHA oversee the districts’ human resource (HR) demands and distribute health workers accordingly. The districts, headed by the district health administrations (DHA) and district hospitals (DH), are responsible for adequately staffing all public health facilities and hospitals within their district. The DHA and DH do not have the authority to hire or fire. Thus, staffing is frequently done by transferring existing staff between facilities, including DHs, health centers, and community-based health planning and services compounds (CHPS). Transfers can take place between facilities within the same district (intra-district), between facilities in different districts within the same region (inter-district), or between facilities in different regions (inter-regional). Transfers can be initiated by health administrators in charge or by health workers.

There is a paucity of research on transfer practices [2, 4, 6]. By gathering perspectives from health administrators with decision-making authority in terms of PT and frontline health workers who have transferred within the public health service delivery agency in Ghana, namely GHS, this exploratory study aims (1) to identify rationales behind transfer decisions and (2) to examine how transfers are managed in practice versus current PT policies and procedures.

## Methods

### Study design

To explore an under-researched topic, this study used a mixed-methods triangulation design combining a structured questionnaire, in-depth interviews, and policy documents. The semi-structured interview guide and questionnaire were developed to explore the processes relating to the transfer of health workers, including the roles and responsibilities of involved health administrators.

### Study setting

This study was conducted in three districts in the Eastern Region of Ghana, including Akwapim North, Kwahu West, and Upper Manya Krobo in 2014 (Table 1). Akwapim North is mostly urban, and Kwahu West is semi-urban, while Upper Manya Krobo is predominantly rural [10]. Upper Manya Krobo has the lowest proportion of health facilities and high-level cadres, including doctors (0.7%), registered nurses (19.4%), and midwives (9%).

**Table 1:** Study district characteristics

	Akwapim North	Kwahu West	Upper Manya Krobo	Total
Population	142 275	97 556	78 158	317 989
Clinical health workforce	231 (37.4)	253 (40.9)	134 (21.7)	618(100)
Doctors	8 (3.5)	9 (3.6)	1 (0.7)	18 (2.9)
Medical assistants	7 (3.0)	3 (1.2)	4 (3.0)	14 (2.3)
Registered nurses	58 (25.1)	71 (28.1)	26 (19.4)	155(25.1)
Midwives	43 (18.5)	31 (12.3)	12 (9.0)	86 (13.9)
Community health nurses	50 (21.6)	53 (20.9)	56 (41.8)	159(25.7)
Auxiliary nurses/Health assistants	46 (19.9)	67 (26.5)	28 (20.9)	141(22.8)
Allied health workers	16 (6.9)	16 (6.3)	6 (4.5)	38 (6.1)
Pharmacists	3 (1.3)	3 (1.2)	1 (0.7)	7 (1.1)
Health facilities	23 (37.1)	27 (43.5)	12 (19.4)	62 (100)
Hospitals	1 (4.3)	1 (3.7)	1 (8.3)	3 (4.8)
Health centers	9 (39.1)	8 (29.6)	5 (41.7)	22 (35.5)
CHPS facilities	13 (56.5)	18 (66.7)	6 (50.0)	39 (59.7)
Main burden of diseases	1. Malaria	1. Hypertension, malaria*	1. Malaria	
	2. Upper respiratory tract infections	2. Malaria, diarrhea*	2. Rheumatism, diarrhea*	
	3. Hypertension, diarrhea*	3. Skin diseases, upper respiratory infections*	3. Anemia	

Information from the time of study in 2014

*Different burden of disease between children and adults; indicates disease among children

### Study population

The study population included health workers, who transferred between 2011 and 2014, and health administrative staff members from the central level (GHS), regional level (RHA), and the district level (DHA and DH), who were involved in transfer decisions. Health workers were invited to participate if they fulfilled the following criteria: (1) worked at a public health facility in one of the study districts at the time of the study and (2) had transferred geographically between facilities between January 2011 and May 2014. Health administrative staff members were invited to participate if they (1) were primarily involved in the management of transfers and (2) worked at the GHS, RHA, or selected DHAs and DH at the time of the study.

### Data collection

Each health worker completed a structured face-to-face survey including closed and open-ended items. The survey gathered socio-demographic and employment information and explored their reasons for transferring and how their transfer was managed, including how they were involved. The health administrators underwent semi-structured in-depth interviews as described by Britten [11]. The interviews concerned their role in managing transfers and reasons for initiating transfers, how health workers were involved, and their perceptions of why health workers’ request transfers. Study participants were interviewed between May and July 2014. The GHS posting policy draft, including guidelines and procedures, was obtained from GHS HR Development Directorate. The obtained document was confirmed relevant in 2018.

### Data analysis

Health worker data were summarized by means, standard deviations, and ranges for continuous variables, and as frequencies and percentages for categorical variables by using Stata (Stata 14; StataCorp LP, College Station, TX, USA). The interviews were transcribed and subsequently coded in the qualitative research software NVivo 11 by using a general inductive approach [12]. In order to analyze the different perspectives and potential discrepancies between practices and policy, we applied a data triangulation approach, as described by Flick, combining the information gathered from administrators, health workers, and PT policy [13].

### Ethical considerations

This study was carried out under the project PERFORM aimed at strengthening health workforce performance [14] under the lead of the Liverpool School of Tropical Medicine (LSTM). Ethical clearance was obtained from the Research Ethics Committee of LSTM (ID No. 12.09), the GHS Ethical Review Committee (ID No. GHS-ERC: 13/05/12), and the Eastern Regional Health Administration. Written informed consent was obtained from all study participants, and personal data were anonymized prior to analysis.

## Results

Overall, an estimated number of 59 frontline health workers met the eligibility criteria for study inclusion. Among those, 44 (74.6%) agreed to participate (Table 2). Non-respondents included health workers who were (1) absent at the day of our visit (*n* = 12) or (2) refused to participate (*n* = 3). A total of 21 health administrative staff members involved in PT processes participated in the study, including eight males. None of the invited administrators rejected participation (Table 3).

**Table 2:** Study district characteristics and socio-demographic information on transferees (*n* = 44)

	Akwapim North (*n* = 24)	Kwahu West (*n* = 8)	Upper Manya Krobo (*n* = 12)	*n* (%)
Age				
Mean (SD)	40.8 (13)	32.4 (9.1)	31.6 (9.3)	36.9 (12.1)
Range	25–59	26–53	23–53	23–59
Sex				
Female	21 (87.5)	4 (50)	7 (58.3)	32 (72.7)
Male	3 (12.5)	4 (50)	5 (41.2)	12 (27.3)
Marital status				
Married	16 (66.8)	4 (50)	5 (41.3)	25 (56.8)
Unmarried	5 (20.8)	4 (50)	7 (58.3)	16 (36.4)
Other	3 (12.5)	0	0	3 (6.8)
Profession				
Doctor	0	2 (25)	0	2 (4.6)
Nurse	2 (8.3)	1 (12.5)	0	3 (6.8)
Community health nurse	12 (50)	4 (50)	9 (75)	25 (56.8)
Midwife	6 (25)	1 (12.5)	0	7 (15.9)
Physician assistant	2 (8.3)	0	1 (8.3)	3 (6.8)
Public health nurse	0	0	1 (8.3)	1 (2.3)
Disease control officer	0	0	1 (8.3)	1 (2.3)
Accountant	2 (8.3)	0	0	2 (4.6)
Educational background				
Certificate	18 (79.2)	5 (62.5)	9 (75)	33 (75)
Diploma	4 (16.7)	1 (12.5)	3 (25)	8 (18.2)
Bachelor	1 (4.2)	1 (12.5)	0	2 (4.6)
Master	0	1 (12.5)	0	1 (2.3)
Years in profession				
Mean (range)	10.2 (2–30)	6.5 (1–23)	5.3 (1.5–10)	8.3 (1–30)
Current facility type				
Health center	14 (58.3)	3 (37.5)	4 (33.3)	21 (47.7)
CHPS	6 (25)	1 (12.5)	3 (25)	10 (22.7)
Hospital	4 (16.7)	4 (50)	2 (16.7)	10 (22.7)
District Health Administration	0	0	2 (16.7)	2 (4.5)
RHC	0	0	1 (8.3)	1 (2.3)
Years at current health facility				
Mean (range)	1.3 (0–3)	0.9 (0–2.5)	1.4 (0–3)	1.3 (0–3)
Years at previous health facility				
Mean (range)	4.6 (0.5–20)	3.2 (1–6)	2.8 (0.5–9)	3.9 (0.5–20)

*CHPS* Community-based Health Planning and Services Facility, *RCH* reproductive and child health

**Table 3:** Interviewed administrators at central, regional, and district levels (*n* = 21)

Level of employment	*n*
Central level	1
Deputy director of human resources in the Ghana Health Service HR directorate	1
Regional level	3
RHA Human resource manager	1
RHA Deputy director of clinical care	1
RHA Deputy director of nursing services	1
District level	9
District director of health services^1,2,3^	3
Human resource officer ^1,2*,3**^	3
Public health nurse ^1,2,3^	3
District hospital	7
Matron deputy^1,3^	2
Human resource officer ^1,3^	2
Administrator^1,3^	2
Medical superintendent ^*3*^	1
Total	21

^1^Akwapim North

^2^Kwahu West

^3^Upper Manya Krobo

*Double function as HR and health information officer

**Interim

Less than one third of the health workers (27.3%) had initiated their transfer themselves (Table 4). The DHA had initiated 65.9% of the transfers, whereas RHA had initiated 6.8% (Table 4). The majority of transfers had taken place intra-district (79.6%). The DHA had initiated most of these (82.9%), whereas inter-district transfers (13.6%) were mostly initiated by the health workers (66.7%) or by the RHA (33.3%). Inter-regional transfers (6.8%) had only been initiated by the health workers (Table 4).

**Table 4:** Type of transfer versus transfer initiated by (*n* = 44)

Type of transfer	Transfer initiated by			Total
	District Health Administration	Health worker	Regional Health Administration	
Intra-district	29 (82.9)	5 (14.3)	1 (2.9)	35 (79.6)
Community health nurse	21	1	0	22
Midwife	5	2	0	7
Physician assistant	2	1	0	3
Nurse	1	1	0	2
Accountant (RHA)	0	0	1	1
Inter-district	0	4 (66.7)	2 (33.3)	6 (13.6)
Community health nurse	2	0	0	2
Accountant (DHA)	0	0	1	1
Public health nurse (DHA)	0	0	1	1
Inter-regional	0	3 (100)	0	3 (6.8)
Doctor	0	2	0	2
Disease control officer (DHA)	0	1	0	1
Total	29 (65.9)	12 (27.3)	3 (6.8)	44 (100)

*DHA* District Health Administration, *RHA* Regional Health Administration

### The rationale behind transfer decisions

The most frequently reported reasons for health workers to initiate transfer from rural to urban areas were marital reasons (i.e., wanting to be closer to their spouse or lack of opportunities for their spouse) followed by a need for easier access to basic services. Rationales behind transfers from urban to rural areas included needs for changed environment and health-related issues. Transfers between rural areas were due to desired vacancies at other facilities, whereas transfers between urban areas were attributed to preferred locations, disagreements with supervisors, or marital reasons (Table 5).

**Table 5:** Geographical direction of health worker transfers versus initiation of transfer (*n* = 44)

Direction of transfer	Transfer initiated by			Total
	District Health Administration	Health worker	Regional Health Administration	
Transfer from rural areas	17 (73.9)	6 (26.1)	0	23 (52.3)
From rural to rural	10	2	0	12 (27.3)
From rural to urban	7	4	0	11 (25.0)
Transfer from urban areas	12 (57.1)	6 (28.6)	3 (14.3)	21 (47.8)
From urban to urban	7	4	2	13 (29.5)
From urban to rural	5	2	1	8 (18.2)
Total	29 (65.9)	12 (27.3)	3 (6.8)	44 (100)

Regional and district administrators’ rationale for initiating transfers of health workers, as demonstrated by the quote below, was based on equal distribution of staff and skills mix according to national HR requirements.

… this facility has more staff than this other…. and because of the staff mix ( …) it is more endowed than the other. So if we move one [health worker] from Facility B to Facility A, Facility A will also come up a bit. (District Director of Health Services)

This practice aligns with the policy guidelines, which states that staff with adequate skills shall be distributed equitably to health facilities based on vacancies and needed skill. Moreover, administrators initiated transfers based on health worker performance, which is assessed via structured annual appraisals. This practice is not explicitly supported by the GHS posting policy or procedures.

### Management of transfers

**Fig. 1:**
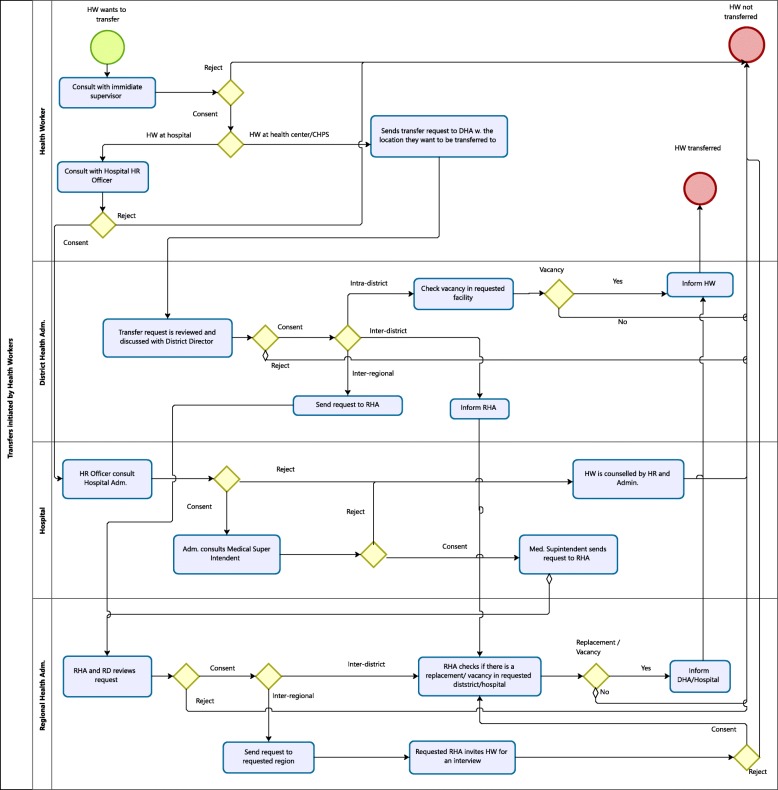
Transfers initiated by health workers

**Fig. 2:**
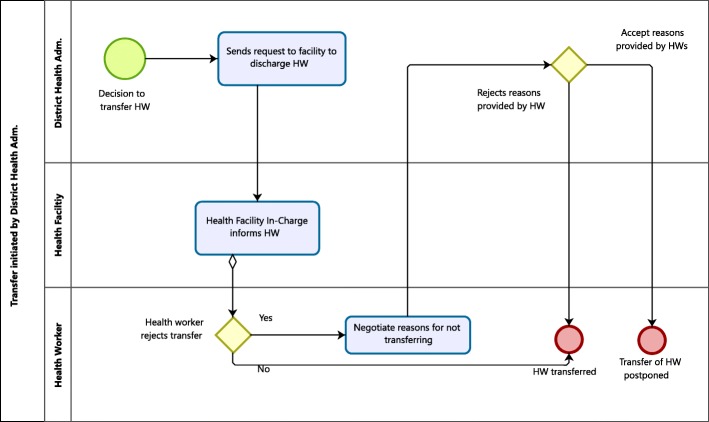
Transfers initiated by the District Health Administration

The management of transfers differed according to the type of transfer (inter-district, intra-district, or inter-regional) and whether the transfer was initiated by the health worker (Fig. 1) or by an administrator (Fig. 2). In order to reflect practice, the figures were derived from information provided by study participants. The figures in general align with the GHS posting procedures, yet take on a health worker-centered approach compared with the procedures’ rigorous focus on bureaucratic measures.

#### Transfers initiated by health workers

As shown in Fig. 1, all transfer types initiated by a health worker relied on consent from the health workers’ immediate supervisor and the District Director of Health Services (DDHS) or the Hospital Medical Superintendent for transfers involving hospital staff. This consent partially depended on the health workers’ ability to convince administrators why a transfer should be granted, cf. quote below.

All they need to do is give us good reasons, just maybe in an informal discussion ( …) why they want to be moved and if the reasons sound good enough we give the approval …. (District Health Administrator)

The policy does not provide input on what qualifies as a “good reason,” yet generically states that transfers can be granted if approved by the Division/Regional Director, if there is a vacancy and if the health worker is not needed elsewhere.

Endorsed intra-district requests for transfer could be effectuated immediately if there was a vacancy at the requested facility. However, inter-district and inter-regional requests had to be passed on from the DDHS to the Regional Director(s) of the involved regions. The Regional Directors had to give their consent and confirm vacancy at the receiving district/region as well as availability of a replacement to fill the void created by the transfer. Our data revealed that inter-district and inter-regional transfers initiated by health workers often were delayed due to ineffective and inefficient means of communication between the districts and the regions, cf. quote below concerning an inter-district transfer.

Depending on how long RHA will need to write to the [other] district to confirm a vacancy, they [the other district] reply, the RHA asks us to release the person, we also reply … (…) Some people when they really want to leave (…) will make sure that he or she brings the letter himself, will sit, pick the response, go back to RHA, sit there, take the response. But if it goes through the normal process … Because when they [RHA] write, they will just put it in a pigeon hole for us (…). So if(…)we have not gone to RHA to pick letters that means(…)the process will be delayed. (District HR Officer)

The posting policy does not include input to guide efficient communication nor state timeframes within which a transfer should be processed.

#### Transfers rejected by transferors

A health workers’ request to transfer could be rejected based on the following: (1) weak reasoning for transfer according to the decision-makers’ discretion, (2) having served insufficient time at current post, (3) recently having received training or professional development beneficial to current post, or (4) having poor performance, cf. quote below. The policy supports the second and third point, but not the first and fourth.

*…* sometimes there are staffs whose output is not too good. So she [the District Director] will know that if that staff goes to her colleague [another District Director] in another place [district], the person will be, let’s say, a sort of nuisance. So she [the District Director] will not prefer someone leaves this place to go somewhere else and gives her a bad name*.* (District HR Officer)

Furthermore, and as indicated previously, transfers could be rejected if there were (5) no vacancies at the requested facility/district or (6) no replacements to fill the health workers’ current position.

A lack of vacancies was not identified as a main cause preventing transfers from happening. However, study participants reported that staff in high supply, such as CHNs, were less likely to be transferred to their preferred location compared with doctors and more senior staff cadres.

A shortage of replacements frequently caused delayed or rejected transfers—especially for health workers in scarce supply, such as midwives, and for health workers placed in rural districts, such as Upper Manya Krobo, where few want to be transferred to. A district public health nurse indicated that a transfer application only would be considered if a replacement had been identified, unless the transfer was “very, very critical,” such as the transferee having a fatally ill family member. The GHS procedures do not define “critical transfers” and do not provide inputs that guide situations where replacements are lacking.

#### Transfers initiated by health administrators

The RHA can initiate transfers of frontline staff at district hospitals, while the DHA has authority to transfer frontline health workers between health facilities (except hospitals) within their district.

When a transfer is initiated by the DHA or RHA (Fig. 2), they send a request to the health workers’ current facility informing them that the health worker has to transfer to a new facility on a given date. The vast majority of health workers reported that neither they nor their facility had been involved in the transfer decision, other than receiving a letter informing them about their transfer. The policy does not state to what extent the health worker and the facilities must be involved. It was reported that health workers could be transferred at any time after having served their required time. Thus, the transfer often came as a surprise to the health worker as well as the facility management, as demonstrated by the quotes below.

When it comes like that, you, the staff or the administrator, you have no idea. You have no objection (…). The letters will come [from the RHA]: “Fine you need to release them before July.” (Hospital HR Officer)

She came back to me: “Why, what have I done? Have I done anything wrong”? So I had to explain things to her that: “We need your service there. That’s why we are transferring you. Not that you’ve done something wrong”. She was here crying. (Health Information Officer)

The latter quote further demonstrates a lack of transparency in the transfer decision process, leaving health workers without an understanding of why they were being transferred.

#### Transfers rejected by the transferee

The posting policy does not provide input for situations where transfers are rejected by a health worker. In practice, it appeared to be difficult for health workers to alter transfer decisions made by DHA or RHA.

… once we have come to the conclusion that this person must go, we will employ all negotiating skills ( …) some will initially resist, but we employ all skills. We talk to the person, give me time, give me this. So we will all sit and … Ok, they have accepted coldheartedly, but there is no choice. (DDHS)

According to the interviewed administrators, transfers are rarely rejected by health workers; most health workers are happy to move, especially those who are being transferred from a rural to urban area. This diverged from the health workers’ responses, where some reported not being happy with the transition, largely because they not were involved in the decision-making process. A few health workers reported a desire to leave their new location or resign earlier for retirement due to their dissatisfaction, while others reported feeling happier after having adjusted to their new environment.

I am alright with the decision since I can’t do anything about it. However, the workload in this facility is so high so I am thinking about resigning to go on early retirement. (Transferred health worker)

Health administrators reported that transfer decisions, on rare occasions, could be postponed if the transferee were able to provide valid reasons for why he or she should stay at their current facility (e.g., health care needs, family obligations, having been placed in a deprived area for a prolonged period) (Fig. 2). Consequences of refusing transfer for hospital staff could be RHA writing a vacation of post and blocking the health workers’ salary.

## Discussion

### Rationales behind transfer decisions

The first objective of this study was to identify rationales behind transfer decisions. The rationales differed according to whether the transfers had been initiated by the health administrators or by the health workers.

#### Transfers initiated by health workers

Transfers initiated by the health workers primarily concerned their family and living conditions; postings in urban areas were generally preferred compared with postings in rural areas. These findings are consistent with other studies in Ghana [7, 15, 16].

The exploration of the rationales behind health workers’ decision to transfer is important, as it provides an insight into why health workers seek to leave their facility. Poor retention of health workers is a significant problem in rural and remote areas, with negative consequences for the delivery of high-quality health services [17, 18].

Literature suggest, in concordance with our findings, that health workers in rural and deprived areas face higher workloads, professional isolation, unsustainable work environments, lack of opportunities for professional advancement, lack of clear contract terms, poor housing, dearth of opportunities, and good schools for spouses and children [15, 16, 19–21]. This firstly makes it challenging to attract health workers to rural areas, as observed in Upper Manya Krobo (Table 1). Secondly, it negatively influences current health workers’ motivation and job satisfaction, which causes them to seek more satisfactory conditions in urban areas, the private sector, or abroad [10, 15, 19, 22]. Bonenberger et al. found that health workers in rural Upper Manya Krobo were five times more likely to leave their current facilities compared with health workers in Akwapim North [10]. Our findings demonstrate that conducive work and living environments, especially in rural areas, play an important role in terms of retaining health workers.

#### Transfers initiated by health administrators

We found that transfers initiated by the DHAs or RHA were based on HR requirements as well as on health workers’ performance. Studies from other LMICs confirm that high-performing health workers frequently are transferred to improve health service performance, while low-performing health workers are likely to stay at their current facility until their output has improved [2, 3]. This practice does not align with the posting principle stated in the GHS policy, namely that staff shall be distributed solely based on vacancies. The lack of explicitly stated transfer procedures allows administrators to initiate transfers based on their own discretion [2, 3, 7, 23]. This may lead to practices that disregard the underlying principle of postings being done under fairness and transparency [2, 24]; low-performing health workers may for example be prevented from improving their output in their existing environment, while being transferred as a result of performing well can be perceived as a punishment rather than a reward [7].

Furthermore, transfers may be initiated as an effort to strengthen local political constituencies, health workers, global health agencies, and community health committees [2, 3]. Studies in LMICs, including Ghana, points out that corruption, including bribery, collusive or personal networks, including nepotism, can underlie transfer practices [2, 3, 6, 7, 23, 25–28]. These patterns were not identified in this study. Our applied data collection method, including recorded face-to-face interviews, may have prohibited study participants from sharing such sensitive information.

### Management of transfers

The second objective of this study was to examine how transfers were managed in practice versus policy. The latter did not provide explicit guidance on several matters, i.e., reasons upon which transfers could be initiated or rejected; to what extent facilities and health workers should be involved in transfer decisions; and timeframes within which transfers should be processed. In practice, transfer decisions appeared to depend on the discretion of the decision-makers, confirmed by other PT studies in Ghana, Uganda, and Nigeria [2, 3, 7, 23]. Moreover, we identified that transfer decisions frequently were made without involving health workers, which led to staff perceptions of an unpredictable and non-transparent PT system, converging with previous findings [3, 7, 16, 27].

As demonstrated in the current study, there are multiple points of negotiation between transferors and transferees. The lack of explicit procedures to guide the negotiations results in transfer decisions that are negotiated outcomes of different preferences and objectives, the administrators’ objectives relating to the needs of GHS versus the health workers’ objectives relating to their individual needs [3]. Due to power dynamics, the administrators are likely to prevail, leaving health workers with unmet needs [7]. The unmet needs facilitate dissatisfaction among health workers, which negatively affects health service delivery, for example by increasing absenteeism [2–4, 7, 25, 29].

A main challenge in HR management is to integrate the needs of the organization with the individual needs of its members [30]. Nevertheless, successful attempts have been made, for example in Zambia, where financial incentives have been successfully applied to motivate health worker transfers to rural areas [18, 22]. The potential of non-monetary incentives has also been demonstrated, i.e., career development, appropriate accommodation, clear terms of appointment with a reliable endpoint, and provisions for the schooling of children [19–21, 31–33]. To our knowledge, such incentives are yet to be implemented in Ghana, despite the skewed distribution of health staff, with less human resources for health in deprived areas [24].

### Proposed recommendations towards a more fair and transparent transfer system

Procedures guiding transfer decisions should be better specified to avoid decisions that are based on the discretion of the individual decision-maker. The procedures should address how the health worker should be involved, guidance on when transfers can be obtained or rejected, and maximum timeframes for transfer processes to reduce communication deficiencies. Further efforts can be considered to ensure fair and transparent transfer processes, such as the ones described by Schaaf and Freedman suggesting that transfer practices become subject to local committee reviews, strengthening outside watch bodies, and creating shared ethical standards [2]. This is supported by Abimbola et al. who suggest having PT decisions reviewed periodically to ensure they are not partial, unfair, or corrupt [3].

### Strengths and limitations of the current study

Strengths of this study include having gathered information and perspectives from both transferred frontline health workers as well as from national-, regional-, and district-level decision-makers. This allowed a more nuanced understanding of PT mechanisms and served as a source of validation of the information on practices collected across the different stakeholders. Limitations of the study include the data collection approach (face-to-face interviews) that may have prohibited attaining sensitive information on informal malpractices. Further exploration of informal practices in regard to PT is called for. Studies exploring PT practices, where informal lobbying and managerial discretion play an important role, may benefit from complementing in-depth interviews and structured interviews with observations to gain an understanding of social networks and informal practices.

Future research may consider a more gender-sensitive approach that differentiates between male and female health workers’ rationales behind transfer and their experiences of transfer. Moreover, additional perspectives of transfer management could be explored by including health workers whose transfer had been rejected, compared with this study that only included transferred health workers.

## Conclusion

We identified a discrepancy in administrators’ and health workers’ rationale and motivation behind transfer decisions. Most health workers desired to be in close proximity to family, professional advancement opportunities, and basic services and did thus not prefer to be transferred to rural deprived areas. However, decision-makers have to distribute health personnel in a way that ensures geographical equity in access to quality healthcare. It is imperative that initiatives are taken to improve rural posts to attract and retain health workers.

Furthermore, this study found that transfer decisions to a high extent relied on the discretion of decision-makers, rather than on formal and explicit procedures. Health workers were often not involved in transfers, and frequently perceived the PT system as unfair and non-transparent. Our policy improvement recommendations aims at making PT policies and procedures in Ghana, and other LMICs, more health worker-centered as health workers essentially are the core of the PT system. Efforts must be made to develop transparent procedures and systems that effectively guide and monitor transfer practices to ensure that these are carried out in a timely, fair, and transparent way.

## Data Availability

The datasets used and analyzed in this study are available from the corresponding author on reasonable request.

## Abbreviations

CHNs: Community health nurses
CHPS: Community-based health planning and services compounds
DDHS: District Director of Health Services
DH: District hospitals
DHA: District health administration
GHS: Ghana Health Service
HR: Human resources
LMICs: Low- and middle-income countries
PT: Posting and transfer
RHA: Regional health administration
RCH: Reproductive and child health
SD: Standard deviation
